# Oro-Anal Transit Time and Colon Manometry: Predictability and Outcomes in Children With Functional Constipation

**DOI:** 10.7759/cureus.48397

**Published:** 2023-11-06

**Authors:** Muhammad Shaukat, Muhammad Altaf

**Affiliations:** 1 Pediatric Gastroenterology, Freeman Health Center, Joplin, USA; 2 Pediatric Gastroenterology, University of Oklahoma Health Sciences Center, Oklahoma City, USA

**Keywords:** ace, colonic manometry, sitzmarks, oro-anal transit time, constipation

## Abstract

Objectives: The primary objective of the study was to evaluate the correlation of oro-anal transit time (OTT) using SITZMARKS® (Konsyl Pharmaceuticals Inc., Easton, MD, USA) with colonic manometry (CM) in children with chronic idiopathic constipation (CIC). The secondary objective was to determine the clinical utility of these studies in directing treatment strategies.

Methods: A retrospective chart review of 44 children with CIC was evaluated with both OTT and CM. The median follow-up was one year (0.3 to 7).

Results: Seventeen children had normal OTT, and 27 had abnormal OTT (slow-transit constipation (STC)). There was no statistical difference between the percentage of children with abnormal CM (13) test results categorized by OTT (23.5% normal OTT vs. 33% abnormal OTT, p = 0.73). A change in therapy was accepted by all 13 children with abnormal CM, but only 26/31 (84%) of the children had normal CM. The CM test results prompted acceptance of treatment change (appendicectomy or medication escalation) in 89% of children. Overall, 31/44 (70%) of children undergoing CM testing had improvement in clinical symptoms. More children with abnormal CM testing improved vs. normal CM but did not reach significance (85% vs. 65%, p = 0.28).

Conclusion: Contrary to previous studies, OTT results did not predict the presence of colonic dysmotility based on colon motility testing. Colonic manometry testing resulted in the acceptance of a change in therapy in approximately 90% of children. More children with colonic dysmotility improved versus those with normal CM studies. Other interventions beyond stimulant laxatives should be considered in children with refractory constipation.

## Introduction

Childhood constipation accounts for 3% to 5% [1,2] of all visits to pediatricians and 10% to 25% of consults with pediatric gastroenterologists [3]. Its incidence is higher than childhood asthma or migraine headache [4] and has a prevalence of 0.7% to 29% [5] with a female-to-male ratio of 2.1:1 [6], but the symptoms are more severe in males [7]. In about 25% of children, symptoms associated with constipation continue into adulthood despite treatment [3]. The direct and indirect healthcare cost of childhood constipation in the United States is estimated at $3.9 billion per year [8]. In about 95% of cases, pediatric constipation has no known organic etiology and is classified as functional or chronic idiopathic constipation (CIC) [9].

Approximately half of the cases of functional constipation are found to have slow transit constipation based on evaluation by oro-anal transit time (OTT), colonic scintigraphy, or a wireless motility capsule study [10,11]. The SITZMARKS® (Konsyl Pharmaceuticals Inc., Easton, MD, USA) study is usually the preferred method to evaluate colonic transit due to its cost-effectiveness, ease of performance, and availability at most centers [11]. Transit studies evaluate the movement of material through the colon but do not evaluate the integrity of the nerves and muscles of the colon or the response of these nerves and muscles to physiologic stimulation (orthocolonic and gastrocolonic reflexes) or pharmacologic stimulation. These responses may have important implications for tailoring appropriate treatment modalities (behavioral, pharmacologic, or surgical) for children with refractory symptoms. Anorectal manometry (ARM) and colonic manometry (CM) studies provide objective neuromuscular assessments of the anal sphincter and colonic motility, respectively [2,12].

To our knowledge, there are only three studies that have evaluated the relationship between OTT and CM [5,10,13]. Tipnis et al. and Dranove et al. used SITZMARKS, and Mugie et al. used scintigraphy to estimate OTT. All three studies reported good sensitivity but poor specificity for the OTT study in predicting the outcome of the CM study. We aimed to study the correlation of OTT (SITZMARKS) with CM in a larger sample size, compare the results of OTT, ARM, and CM, and determine the outcomes of treatment changes that were made based on the results of CM.

## Materials and methods

**Figure 1:**
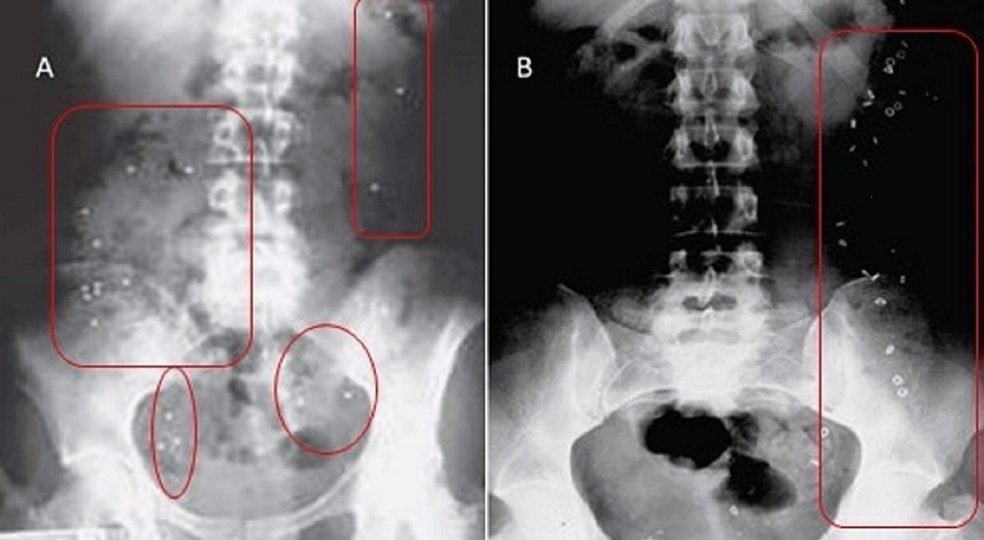
SITZMARKS study A: Diffuse STC, i.e., SITZMARKS is spread throughout the colon as marked; B: Left-sided STC, i.e., SITZMARKS is localized mainly in the left colon as marked STC: Slow-transit constipation

We performed a retrospective chart review of 44 children (Table 1) with a diagnosis of CIC who underwent evaluation with OTT (SITZMARKS) and CM from July 2010 to December 2017 at a children’s hospital. This study was approved by the University of Oklahoma (approval no. 2917). Chronic idiopathic constipation was defined according to the Rome III criteria for children. The OTT was assessed by using the 24 radiopaque markers (SITZMARKS). According to the protocol, children were advised to ingest the markers on day 0 of the study, and abdominal radiographs were done on days 3 and 5 of the study. The information on the SITZMARKS was collected from the radiology reports as well as a review of the X-rays by the author. Fecal impaction, if present, was cleared a week prior to the initiation of the SITZMARKS study. Patients were instructed to take a clear liquid diet and avoid solid foods for at least one day during the bowel cleanout process. Parents and children were instructed not to use laxatives, enemas, or suppositories for the duration of the study. Abnormal OTT was defined as >25% retained radiopaque markers in the colon on day 5 (as seen on an abdominal X-ray) [10,13]. The abnormal OTT study was subcategorized as diffuse slow-transit constipation (STC), i.e., SITZMARKS randomly scattered throughout the colon, or left-sided STC (SITZMARKS in the left colon only) (Figure 1).

**Table 1:** Characteristics of the participants OTT: Oro-anal transit time

Characteristics	Normal OTT (N=17)	Abnormal OTT (N=27)
Sex	Male (N=9), Female (N=8)	Male (N=11), Female (N=16)
Constipation (N) (%)	17 (100)	27 (100)
Encopresis (N) (%)	3 (15)	7 (33)
Abdominal pain (N) (%)	5 (26)	4 (14)
Vomiting/reflux (N) (%)	4 (21)	3 (11)
Failure to thrive (N) (%)	1 (5)	2 (7)

For CM, all the patients were started on a clear liquid diet one day before testing, made nil per os for at least eight hours before the study, and had a bowel cleanout using polyethylene glycol 3350 one day prior to the CM procedure. Drugs with an effect on colonic motility, such as ondansetron, stimulant medications, and anticholinergic and antidepressant drugs, were stopped three days before the study. Under general anesthesia (GA), a colonoscopy was performed, and a motility catheter was placed over the guidewire into the cecum using fluoroscopy. We specifically made sure that no patient was given narcotics, paralytics, alpha/beta agonists and antagonists, ondansetron, or benzodiazepines during GA. We used a 14Fr, unidirectional solid-state high-resolution manometry (HRM) catheter (Unisensor; Medical Measurement Systems, Enschede, Netherlands), which has 36 sensors, with each sensor 3 cm apart.

After two hours of recovery time from GA, each study was done over a six-hour period: two hours of fasting followed by a high-calorie meal (~ 20 kcal/kg), one hour of recording after the meal, then bisacodyl administration (single dose 0.2 mg/kg, max 10 mg) via CM catheter with a further two hours of recording. For consistency in interpreting the results, all of the CM and ARM studies were interpreted by one of the authors, a specialist in colonic motility disorders [13]. Normal CM was defined as the presence of high-amplitude propagating contractions (HAPC) of at least 60 mmHg that propagate >30cm from the proximal colon to the distal sigmoid colon [10]. The CM was considered abnormal if no HAPC were seen after stimulation with bisacodyl. Colon motility was also evaluated for abnormal motor patterns (simultaneous, low-amplitude, retrograde, and uncoordinated contractions).

In our study, ARM was done using high-resolution catheters on all the patients undergoing CM. Under GA, the catheter was placed into the rectum, and the balloon was straightened using the pull-through method. The two most important components of ARM in our study were resting anal pressure and recto-anal inhibitory reflex (RAIR). Once the optimal position of the anal sphincter had been located, resting anal pressure was recorded over a minimum of 30 seconds (normal 83 + 23 mm Hg). High resting anal pressure may indicate anal achalasia and low pressures are usually seen in patients who have undergone anorectoplasty for anal malformations. For evaluation of RAIR, the balloon was inflated rapidly with 5 ml to 10 ml volume increments (maximum insufflation was up to 250 ml) and relaxation of internal anal sphincter pressure was noted. A RAIR is considered to be present if the pressure decreases by at least 5 mmHg [12]. The RAIR can be falsely affected by a decrease in the resting anal pressure due to certain anesthetics used, such as propofol and glycopyrrolate [12]. At our center, we avoid the use of drugs that decrease the resting anal pressure while performing RAIR. Statistical analysis was done using Fisher's exact test to calculate sensitivity, specificity, p-value, and 95% CI, and Cohen's Kappa was used to calculate the correlation between the OTT and CM studies.

## Results

Of the 44 children (two to 17 years old), 55% were females (median age of 9.5 years). The median duration of follow-up was one year (0.3-7). Other reported symptoms included fecal incontinence (10, 23%), reflux (7, 16%), abdominal pain (9, 20%), and failure to thrive (3, 7%). These characteristics were not statistically significant between children with normal and abnormal OTT studies (as seen above in Table 1).

Findings

**Figure 2:**
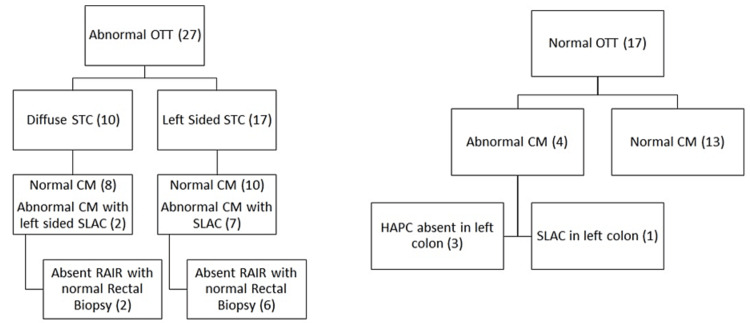
Result of CM in patients with normal and abnormal OTT CM: Colon manometry, OTT: Oro-anal transit time, SLAC: Simultaneous low amplitude contractions, RAIR: Recto-anal inhibitory reflex, HAPC: High-amplitude propagating contractions

Transit marker studies were normal in 17 (39%) and abnormal in 27 (61%) children. In children with abnormal OTT studies, 17 (63%) had a predominantly left-sided marker distribution, and 10 (37%) had a diffuse marker distribution (Figure 2). Colon motility testing was normal in 31 (70%) children and abnormal in 13 (30%). The abnormal CM findings observed were an absence of colonic contractions in the left colon (3, 23%) and non-propagating low-amplitude contractions in the sigmoid colon (10, 77%). Anorectal manometry was normal in 36 (82%) and abnormal in 8 (18%). In all children with an absence of anorectal sphincter relaxation, rectal biopsies were normal.

Comparison of transit markers with ARM and CM testing results

Comparisons of OTT Studies With ARM Studies

Around 30% of children with abnormal OTT studies had an abnormal ARM, compared to none of the children with normal OTT studies (p = 0.016). The sensitivity, specificity, positive predictive value (PPV), and negative predictive value (NPV) of the OTT test results to predict ARM test results were 30%, 100%, 100%, and 47.2%, respectively. Similarly, the correlation between OTT and ARM results was weak (ƙ=0.245, p = 0.013).

Comparison of OTT Studies With CM Studies

In children with normal OTT studies, 13/17 (76%) had normal CM test results, while 4/17 (24%) had abnormal CM test results with absent colonic contractions in the left colon (three) and non-propagating low-amplitude contractions in the sigmoid colon (one). In children with abnormal OTT studies, 18 (67%) had normal CM test results, and nine (33%) had abnormal CM test results. There was no statistical difference in the frequency of abnormal CM studies between children with normal and abnormal OTT studies (p = 0.7345). The ability of an OTT study to predict an abnormal CM study was poor: sensitivity at 33%, specificity at 77%, PPV at 70%, and NPV at 42%. The correlation of the OTT study with the CM study was low (ƙ=0.085, p=0.488).

Comparison of ARM Studies With CM Studies

Of the 36 children with normal ARM studies, 30 had normal CM studies, and six had abnormal CM studies: absent colonic contractions in the left colon (three) and non-propagating sigmoid low amplitude contractions (three). Of the eight children with abnormal ARM studies, one had normal CM studies, and seven had abnormal CM studies. There was a statistical difference in the frequency of abnormal CM studies between children with normal and abnormal ARM studies (p<0.001). The ability of an abnormal ARM study to predict an abnormal CM study was fair (sensitivity (88%), specificity (83%), PPV (54%), and NPV (97%)), and the correlation of the ARM study with the CM study results was moderate (ƙ=0.57, p<0.001).

Comparison of Combined OTT and ARM Study Results With CM Study Results

In children with both normal OTT and ARM studies (17 children), 13 (76%) had normal CM studies and 4 (24%) had abnormal CM studies (Figure 2). In children with abnormal OTT and normal ARM studies (19 children), 17 (89%) had normal CM studies, and two (11%) had abnormal CM studies. In children with both abnormal OTT and ARM studies (eight children), one (12%) had normal CM studies and seven (88%) had abnormal CM studies. No children had a combination of an abnormal ARM and a normal OTT study. The additional finding of an abnormal ARM study in a child with an abnormal OTT study improved the ability to predict an abnormal CM test result (specificity 88%, sensitivity 90%, PPV 78%, NPV 94%, p<0.001). Overall, the correlation of combined OTT and ARM testing with CM testing results was slight (ƙ=0.098, p>0.05). However, in children with abnormal OTT studies, the correlation of ARM testing with CM testing results was good (ƙ=0.743, p<0.001).

Patient outcomes

Clinical improvement was defined as at least a 50% increase in the number of stools per week and a decrease in the number of stool accidents from baseline. Overall, 39 (89%) children (13 abnormal CM, 26 normal CM) had a change of therapy after motility testing. Twenty-six (59%) children accepted escalation of medication therapy (increasing the dosage of the previously used osmotic and stimulant laxatives with or without the addition of another laxative), 12 (27%) received antegrade continence enemas (ACE) after an escalation of medication therapy, one (3%) received ACE immediately after testing, and five (11%) did not accept a change in therapy.

More children who accepted a change in therapy improved (29, 74%) compared to children who did not accept a change of therapy (2, 40%) but did not reach statistical significance (p = 0.144). More children accepting ACE improved compared to all others (12 (93%) vs. 19 (61%), p = 0.68), and those receiving medication escalation alone (17 (65%), p = 0.12), but did not reach statistical significance (Table 2).

**Table 2:** Patient outcomes CM: Colonic manometry, ACE: Antegrade continence enema A p-value is significant if <0.05.

Result of CM	ACE	Medication Escalation	No Change in Therapy	Clinically Improved: N = 31/44 (70%)
Abnormal (N = 13)	N = 6/6 improved	N = 5/7 improved	-	N =11/13 (85%) (p = 0.28)
Normal (N = 31)	N = 6/7 improved	N = 12/19 improved, N = 1 lost to follow-up	N = 2/5 improved, N = 3 lost to follow-up	N = 20/31 (65%)

## Discussion

This study focuses on the utilization of the OTT study (SITZMARKS) in predicting the results of the CM study in children with CIC and the outcome of treatment changes made after the CM study. There is a paucity of literature on this topic, and only three studies have previously looked at the relationship between the OTT study and the CM study [5,10,13]. In the first study by Tipnis et al., radiopaque markers were used to estimate CTT; all five participants with normal OTT had normal CM and only nine out of 19 participants with abnormal OTT had abnormal CM. However, in this study, CM was considered normal if HAPC propagated at least 30 cm without further definition of the termination landmark of the colon, such as the transverse, descending, or sigmoid colon. In the second study by Mugie et al., colonic scintigraphy was used to estimate the colonic transit time (CTT), and 14 out of 26 participants showed similar results in colonic scintigraphy and manometry. The overall sensitivity of scintigraphy was 0.8, and the k score was 0.34, which shows a fair agreement between the two studies. Dranove et al. reported that 18.5% (5/27) of children with abnormal OTT had normal CM, and 85.7% (6/7) of children with normal OTT had normal CM. They also reported that about 73% of the study group underwent surgery for further management of the constipation.

To date, our study is the largest to evaluate the role of motility testing in children with CIC, comparing the results of the OTT, ARM, and CM and determining the outcomes of treatment changes that were made based on the results of CM. Our study is distinct from other studies in several ways. First, in contrast to previous studies, this study revealed a poor correlation between the OTT study and the CM study, and OTT is neither sensitive nor specific in predicting the outcome of the CM study. Second, the absence of a RAIR in ARM studies had a good correlation with abnormal distal colonic motility. This finding was not highlighted in other studies. Third, the best clinical outcomes occurred in children who accepted antegrade enemas, highlighting the fact that CM should be pursued in children who fail to respond to conventional medical treatment even if an OTT study reveals normal results.

Children with STC can be further assessed by OTT studies such as SITZMARKS, colonic scintigraphy, or wireless motility capsules. The SITZMARKS study is considered the standard method due to its cost-effectiveness and easy availability at most centers, but the difficulty in accurately visualizing and locating these markers on abdominal radiographs makes it vulnerable to error counting and inter-observer variability [5,14,15]. To minimize these risks, we divided the colon into only two segments (proximal to splenic flexure and distal to splenic flexure) as opposed to the right colon, left colon, and recto-sigmoid (Arhan method). On the other hand, a scintigraphy study requires more expertise, is expensive, and poses the risk of excessive radiation exposure. Although there are no pediatric studies comparing the SITZMARKS with scintigraphy to determine the OTT [10], adult studies have shown comparable results of the two modalities in estimating the OTT [16,17]. Wide variations exist in reporting the CTT in healthy and constipated children, depending on the methodology used [1]. Velde et al. reported a median total CTT of 36 hours (<2.4 to 86.4 hours) in 54 healthy children between three and 18 years of age [14].

Both segmental and propagative colonic contractions are more likely to be deranged in children with STC [10,18], with recto-sigmoid and the descending colon as the most commonly affected areas [19]. This is consistent with our results, as all of our abnormal CM studies showed left-sided colonic dysmotility even in children with diffuse STC on the OTT study. Of the four children with normal OTT but abnormal CM, three had absent HAPC in the left colon and one had non-propagating sigmoid low amplitude contractions (SLAC) in the left colon, indicating a neuromuscular pathologic process. Although not fully understood, the disparity between the OTT study and CM results can be explained by the fact that CM is performed after bowel cleanout, and bisacodyl is used to induce HAPC, which is not the case with the OTT study [5]. Moreover, SITZMARKS, being a non-food and non-digestible particle, may move differently from food in the gastrointestinal (GI) tract and therefore not reflect the true physiologic motility pattern.

Eight of our patients with abnormal OTT study and SLAC had absent RAIR. Hirschsprung’s disease was ruled out in these children with a normal rectal biopsy. An overstretched colon secondary to CIC gradually develops deranged contractility of the dilated segment with the absence of RAIR and the complete absence or early termination of HAPC. Koppen et al., who studied the relationship of left colonic diameter on barium enema and colonic manometry in a group of 30 children with intractable functional constipation, reported that children with premature termination of HAPC prior to the left colon had significantly larger colonic diameters than those who have fully propagated HAPC [20]. Moreover, the left colonic diameter was >6.5 cm in half of the children, which is the cut-off to define megacolon or megarectum in adult patients. Pensabene et al. also reported simultaneous low-amplitude contractions in the distal dilated colon in 17% of their study participants who were undergoing a CM study for intractable lower GI symptoms [2]. Some authors have stated that the standard CM protocol that is being used in most centers may be overestimating patients with colonic dysmotility due to several factors. Borrelli et al. reported that using the higher dose of bisacodyl (0.4 mg/kg) during a CM study causes a significant increase in HAPC compared with the standard dosing of 0.2 mg/kg [21], thus decreasing the number of abnormal CM studies. Others talk about the effects of general anesthesia on the findings of colonic motility [12,22]. Arbizu et al. noted a significantly higher motility index, number of HAPC, and proportion of patients having HAPC on day 0 of CM compared to day 1 of CM. Moreover, 47% of the patients with an abnormal CM study on day 1 changed to a normal study on day 2, and others showed improvement in the number and propagation of HAPC. The author concluded that CM parameters are affected by GA and possibly by colonic manipulation during colonoscopy [22]. However, further large-scale studies are needed to modify the current motility protocol, which states that a CM study can be performed after at least two hours of full recovery from anesthesia [12].

The clinical utilization of CM in determining the patients who would potentially benefit from surgical intervention (appendicectomy vs. ileostomy vs. surgical resection of the affected segment) or reanastomosis of the diverted colon has been addressed in several studies [2,12,23]. According to a survey study by Koppen et al., out of all the pediatric surgeons and pediatric gastroenterologists who responded to the survey, 38% were using the CM study in children with intractable constipation, 61% for differentiating myopathic vs. neuropathic dysmotility, and 57% for surgical decision-making [6]. Rodriguez et al. studied 40 individuals with chronic constipation who had an abnormal CM study and subsequently underwent ACE. A repeat CM was performed to evaluate the effect of ACE. Out of these 40 children, 33% showed normalization of their CM study after undergoing ACE, and 28% were able to successfully discontinue ACE after a median duration of 19 months [23]. In our study, 92% of the children who underwent ACE showed clinical improvement in their symptoms.

Why children with normal CM continue to have intractable constipation despite treatment is not completely understood; the other colonic motor patterns and extrinsic parasympathetic input to the colon may be abnormal in these children [24]. The main limitation of our study is its retrospective nature, and we do not know if all the children strictly followed the standard SITZMARKS protocol for OTT studies. Moreover, counting and localization of SITZMARKS are observer-dependent; therefore, there is always the possibility of inter-observer variability.

## Conclusions

An OTT study is not truly reflective of colonic motility in patients with CIC and should not be used anymore for this purpose. Instead, a CM study should be pursued in patients who do not respond to standard medical therapy. We believe the CM study offers therapeutic benefits, as 89% of the children in our study who had CM testing accepted the change in therapy. We also believe that, besides medication escalation, other interventions such as ACE should be considered in children with refractory constipation. Future prospective studies using standardized protocols and a larger number of study participants may be needed to further explore these findings.
